# A New Host–Parasite Association: A *Glugea* sp. (Microsporidia) Infecting the Commercial Fish *Sardina pilchardus* from an Atlantic Fishery

**DOI:** 10.3390/life16050733

**Published:** 2026-04-28

**Authors:** Enrique Baquero, Gabriel Reina, Rafael Jordana

**Affiliations:** 1 Institute for Biodiversity and Environment BIOMA, University of Navarra, 31008 Pamplona, Spain; ebaquero@unav.es; 2 Department of Environmental Biology, School of Sciences, University of Navarra, Irunlarrea, 1, 31008 Pamplona, Spain; rjordana@unav.es; 3 Department of Microbiology, Clínica Universidad de Navarra, Avda. Pío XII, 32, 31008 Pamplona, Spain; 4 IdiSNA, Navarra Institute for Health Research, 31008 Pamplona, Spain

**Keywords:** 18S rRNA, commercial fish parasite, *Glugea* sp., molecular taxonomy, One Health, spore morphology

## Abstract

A microsporidian species, *Glugea* sp., was identified infecting the visceral cavity of commercial sardines (*Sardina pilchardus*) from an Atlantic fishery. Macroscopic examination revealed a white, friable mass that readily disintegrates, releasing spherical to ovoid microsporidian spores. Ultrastructural analysis shows an isofilar polar tube with 10–15 coils arranged in one to three rows. The study combined macrophotography, light microscopy, histological staining, transmission electron microscopy (TEM), and molecular analyses. Morphologically, *Glugea* sp. shares some features with *Glugea vincentiae;* however, molecular data do not support a close phylogenetic relationship between these two species. Phylogenetic analysis based on 18S rRNA sequences places the organism within a clade comprising *G. plecoglossi*, *G. thunni*, *G. atherinae*, *G. gasterostei*, *G. hertwigi* and *G. anomala*. Despite the high sequence similarity observed within this group, morphological and ultrastructural characteristics allowed differentiation of the present species, highlighting the limitations of relying solely on conserved molecular markers for species delimitation. A comprehensive morphological and molecular description of *Glugea* sp. is provided. Given the ecological and economic relevance of microsporidia, as well as their recognized role in animal and human disease, this new host–parasite association may have implications for fisheries and food safety, particularly considering the widespread consumption of sardines.

## 1. Introduction

Microsporidia (Phylum Microsporidia Balbiani, 1882) are a diverse group of obligate intracellular parasites that infect a wide range of hosts, including arthropods and vertebrates. These unicellular eukaryotes are frequently reported in fish, affecting both wild populations and species reared in aquaculture and aquarium systems [1,2,3,4,5,6,7]. In commercially important species, microsporidian infections can have significant impacts on production, product quality, and food safety [8,9,10,11,12,13,14,15,16]. In recent years, increasing attention has been paid to the ecological role of Microsporidia and their involvement in emerging diseases affecting aquatic organisms [17,18,19,20], as well as their potential relevance to human health, particularly among immunocompromised individuals [21].

Members of the genus *Glugea* are considered cosmopolitan organisms [22,23], characterized by the formation of xenomas, which possess a thick wall composed of fibrous material and concentric fibroblasts. Xenoma development is highly specific, typically occurring in particular organs and involving connective tissue cells [24,25]. Despite their relevance, key aspects of host–parasite interactions within the Glugeidae, as well as detailed spore morphology—critical for species identification—remain insufficiently documented [17].

The emergence of a novel parasite–host association is especially noteworthy when it involves commercially important fish species, particularly those taken from open marine environments where infection control is inherently difficult. In such contexts, reliable identification of the causative agent demands a combined assessment of infection patterns, ultrastructure, and molecular data. In this study, we describe an infection observed in sardine (*Sardina pilchardus* (Walbaum, 1792)), characterized by the presence of whitish vesicles of 5–20 mm in the visceral cavity of the fish.

To date, microsporidian infections have not been reported in sardines, with the exception of an early description from 1895 referring to *Glugea cordis* in *Alosa sardina*, a species distinct from *Sardina pilchardus* [26]. However, infections have been documented in other members of the family Clupeidae (*Alosa* Linck, 1790 and *Dorosoma* Rafinesque, 1820) [27,28,29]. Considering the pelagic nature of sardines, their plankton-based diet, and their widespread consumption, the identification of microsporidian infections in this species may have important implications for both fisheries and food safety.

A major challenge in the taxonomy of *Glugea* lies in the frequent occurrence of high similarity among SSU rRNA sequences across species that nonetheless exhibit distinct morphological and biological features. Although molecular data are essential, they do not always provide sufficient resolution for species delimitation in this group. In cases where the complete intracellular life cycle cannot be fully characterized, morphological and ultrastructural features, together with host and infection characteristics, remain critical for distinguishing taxa. Within this framework, a novel host–parasite association involving *Sardina pilchardus* is described in detail, and its ecological and economic implications are examined.

## 2. Materials and Methods

Several infected sardines, captured in an Atlantic fishery, were identified during routine commercial processing prior to distribution and subsequently submitted to the laboratory for further investigation. Given the unknown infectivity of the observed organisms in the sardines, enhanced biosafety measures were implemented throughout sample handling. Unfixed material was incinerated, and all working areas were treated with methanol, following published fixation-based procedures in which methanol is employed to inactivate microsporidian spores.

**Figure 1:**
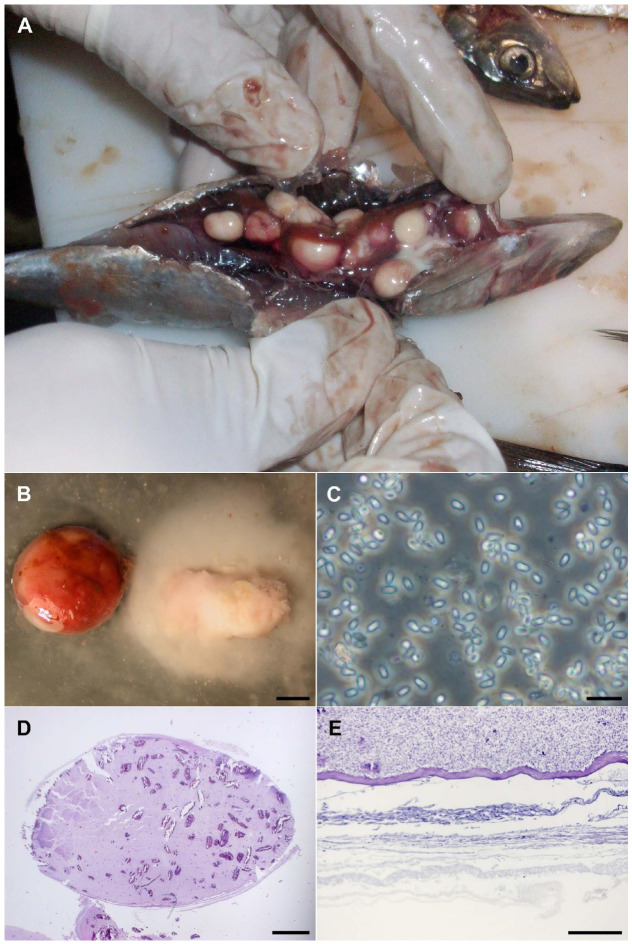
Morphology of the infection caused by *Glugea* sp. (**A**) Macro photograph of commercial sardine infected with black-appearing cysts. (**B**) Close-up of the cysts, one untouched and one with the contents spilled (bar 2 mm). (**C**) In vivo spores in a smear (bar 10 micrometers). (**D**) Cross section of a whole cyst, stained with Hematoxylin–Eosin (bar 1 mm). (**E**) Detail of the external part of the cyst, showing the layer of fibroblasts, PAS tinction (bar 100 micrometers).

Multiple specimens were examined, and between 2 and 10 soft cysts per fish were aseptically collected from the visceral cavity (Figure 1A). These cysts appeared as white, soft structures with a thin and highly fragile outer layer (Figure 1B). Fresh samples were stored in fish physiological saline solution (0.9%) until further processing. Additional cysts were preserved in 100% ethanol, 4% formaldehyde or frozen at −30 °C until molecular and ultrastructural investigation was performed.

### 2.1. Optical Microscopy

For initial light-microscopy examination, slides of the fluid inside the cysts were wet-mounted (Figure 1C) or fixed with 100% methanol for Giemsa staining. Then, following paraffin fixation of the cysts, slides for Hematoxylin-Eosin (HE) (Figure 1D), Giemsa and Periodic Acid-Schiff (PAS) stains were prepared. The observation of the slides showed a cell layer surrounding the group of spores within each cyst (Figure 1E), but no septa or circulatory tissue were observed. Microscopic examination revealed an infestation caused by Microsporidia.

### 2.2. Electron Microscopy

Samples for electron microscopy were fixed with 4% (*v*/*v*) glutaraldehyde in 0.1 M cacodylate buffer (pH 7.3) for eight hours. The samples were maintained for 24 h in a 0.25 M sucrose buffer containing 0.1 M cacodylate and post-fixed in 1% (*w*/*v*) osmium tetroxide in phosphate buffer (pH 7.4) for 2.5 h. The samples were then dehydrated in ethanol and embedded in Epon resin.

Epon embedding was done in six stages: (i) 10% (*v*/*v*) Epon + 90% (*v*/*v*) propylene oxide for 18 h; (ii) 25% (*v*/*v*) Epon + 75% (*v*/*v*) propylene oxide for 9 h; (iii) 50% (*v*/*v*) Epon and 50% (*v*/*v*) propylene oxide for 15 h; (iv) 75% (*v*/*v*) Epon + 25% (*v*/*v*) propylene oxide for 5 h; (v) 90% (*v*/*v*) Epon + 10% (*v*/*v*) propylene oxide for 4 h; and (vi) 100% Epon. The resin was then polymerized in an oven with the temperature slowly increasing from ambient temperature to 60 °C within 48 h to ensure proper polymerization: first raising the temperature from ambient to 37 °C in one hour, followed by incubation at 37 °C for 15 h; next raising the temperature from 37 to 45 °C in one hour, followed by incubation at 45 °C for 15 h; and finally raising the temperature from 45 °C to 60 °C in one hour, followed by incubation at 60 °C for 15 h.

**Figure 2:**
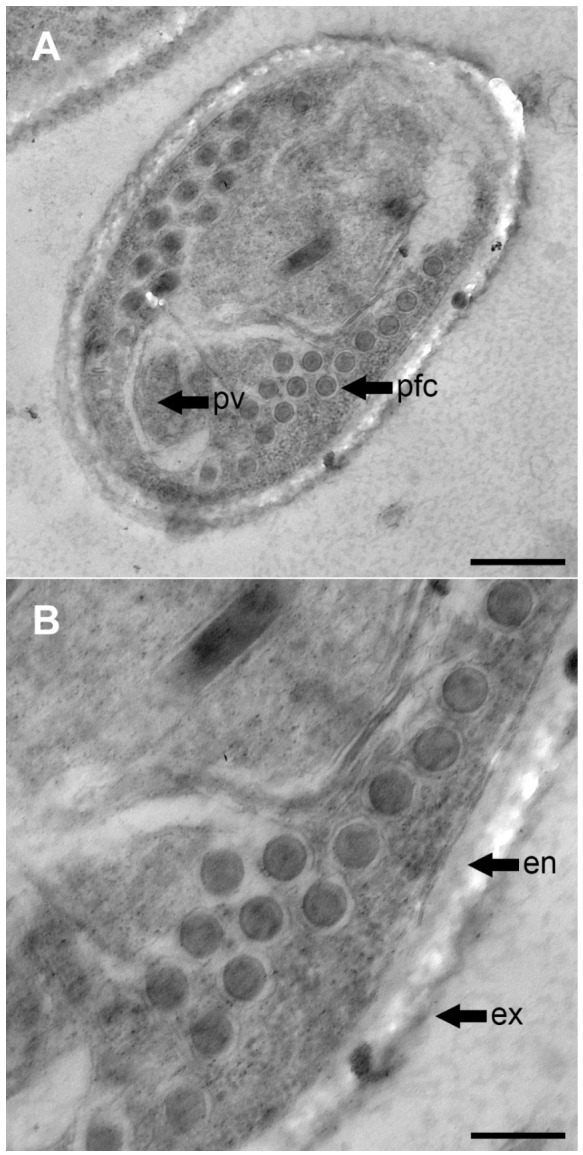
TEM photographs of the spore of *Glugea* sp. ex sardine. (**A**) Cross section of a mature spore showing the arrangement of the coils is an isofilar type and rows (bar 500 nm). (**B**) Filaments composed of six layers (bar 200 nm). pv, posterior vacuole; pfc, polar filament coils; en, endospore; ex, exospore.

Semi-thin sections were stained with toluidine blue and evaluated by light microscopy. Selected ultra-thin sections were observed using a Zeiss LIBRA 120 transmission electron microscope (Zeiss, Oberkochen, Germany) (Figure 2A,B).

### 2.3. DNA Extraction, Amplification, and Sequencing

DNA was extracted using QIAamp^®^ DNA Mini Kit (Qiagen, Hilden, Germany). A two-step process was employed to eventually get the sequence of the region coding for the small subunit (SSU) rRNA gene of this microorganism. First, the region coding for the large subunit (LSU) ribosomal RNA (23S rRNA) was amplified using two generic microsporidian primers [30] for this target (NAP_99: 5′-AACAGRTCMGWKATGCCCT-3′ as forward primer, and 580R: 5′-GGTCCGTGTTTCAAGACGG-3′ as reverse primer), to get a 768 bp amplicon closely related with *Glugea anomala* (Moniez, 1887) (AF056016) and *Glugea stephani* (Hagenmuller, 1899) (AF056015) after sequencing and BLAST analysis (NCBI, https://blast.ncbi.nlm.nih.gov/; accessed on 10 February 2026).

Using the LSU sequence as a basis for primer design, the region coding for the SSU (18S rRNA) was amplified in three overlapping fragments: forward primer MICROF and reverse primer MICRO_R451 for fragment 1; MICRO_F426 and MICRO_R875 for fragment 2; and MICRO_F831 and 1492N [7] for fragment 3. The three resulting amplicons (470 bp, 450 bp, and 406 bp, respectively) were purified and sequenced using the primers described in Table 1.

All, LSU and SSU PCRs were carried out in 50 µL reaction volume, with 20 pmol of each primer, 10 nmol of each dNTP, 2 mM MgCl_2_, AmpliTaq Gold Buffer 1×, 1.5 U DNA polymerase (Applied Biosystems, Foster City, CA, USA). Amplifications were run on an Applied Biosystems 4600 Thermocycler (Applied Biosystems, Foster City, USA) with initial polymerase activation for 10 min at 95 °C, followed by 45 cycles of denaturation for 30 s at 95 °C, annealing for 30 s at 65 °C, and extension for 75 s at 72 °C. Final extension was done at 72 °C for 10 min, followed by a hold step at 4 °C. The amplification products were purified using Illustra GFX PCR DNA and Gel Band Purification Kit (GE Healthcare, Buckinghamshire, UK) to eliminate unincorporated primers and dNTPs.

Both strands of the PCR products were sequenced by using the same primers that were used for amplification. Sequencing reactions were performed at CIMA Genomics Core Facility, University of Navarra with the ABI PRISM BigDye Terminator Cycle Sequencing Kit (Applied Biosystems) and reactions were analyzed on the ABI 3700 automatic DNA sequencer (Applied Biosystems). The resulting sequences were assembled and manually corrected using Clustal Omega (EMBL-EBI web server; accessed on 10 February 2026; http://www.ebi.ac.uk/Tools/msa/clustalo/). Finally, a consensus sequence of 1334 bp for the 18S rRNA gene was obtained from three independently amplified and sequenced products.

### 2.4. Distance and Phylogenetic Analysis

To evaluate the phylogenetic relationship of the organism described and other microsporidians, a selection of 48 related microsporidians was based on BLAST homology scores and the availability of complete SSU rRNA sequences. SSU rRNA sequences used and NCBI accession number obtained from Genbank were as follows: *Ameson herrnkindi* (MN935433); *Cambaraspora* sp. (MT006314); *Cucumispora dikerogammari* (GQ246188); *Dasyatispora levantinae* (GU183263); *Dictyocoela cavimanum* (AJ438959); *Facilispora margolisi* (HM800849); *Glugea anomala* (AB923879); *Glugea anomala* (AF044391); *Glugea arabica* (KT005391); *Glugea atherinae* (U15987); *Glugea epinephelusis* (AY090038); *Glugea gasterostei* (KM977990); *Glugea hertwigi* (GQ203287); *Glugea nagelia* (KJ802012); *Glugea plecoglossi* (AB623035); *Glugea plecoglossi* (OR722585); *Glugea plecoglossi* (OR733697); *Glugea serranus* (KU363832); *Glugea thunni* (OM914139); *Glugea* sp. (MT680621); *Heterosporis* sp. (KC137548); *Heterosporis anguillarum* (AF387331); *Ichthyosporidium weisii* (JQ062988); *Ichthyosporidium* sp. (L39110); *Inodosporus octosporus* (MH911629); *Loma acerinae* (AJ252951); *Loma embiotocia* (AF320310); *Loma psittaca* (FJ843104); *Loma salmonae* (HM626203); *Loma* sp. (AF104081); *Microsporidium cerebralis* (JQ316511); *Microsporidium* sp. (AY140647); *Myosporidium merluccius* (AY530532); *Nucleospora salmonis* (U78176); *Pleistophora hyphessobryconis* (KM458272); *Pleistophora mulleri* (FN434084); *Pleistophora typicalis* (AF044387); *Pleistophora* sp. PA (AJ252958); *Pleistophora* sp. 3 (AF044390); *Pleistophora* sp. KB-2011 (JN575482); *Pleistophora mirandellae* (AJ252954); *Ovipleistophora mirandellae* (AF356223); *Ovipleistophora ovariae* (AJ252955); *Potaspora morhaphis* (EU534408); *Pseudoloma neurophilia* (AF322654); *Spraguea gastrophysus* (GQ868443); *Thelohania butleri* (DQ417114); *Trachipleistophora hominis* (AJ002605); *Triwangia caridinae* (JQ268567) and *Vavraia culicis* (AJ252961).

These sequences were retrieved from the GenBank database (https://www.ncbi.nlm.nih.gov/genbank; accessed on 10 February 2026) and aligned with the 18S rRNA sequence of *Glugea* sp. specimens found in the present study by using the CLUSTAL Omega website [31,32]. Phylogenetic and molecular evolutionary analyses were conducted using MEGA version 11 [33,34,35]. The evolutionary history was inferred by using the Maximum Likelihood method based on the Tamura-Nei model [36].

A bootstrap consensus tree inferred from 10,000 replicates, represents the evolutionary history of the taxa analyzed. Initial tree(s) for the heuristic search were automatically obtained by applying Neighbor-Join and BioNJ algorithms to a matrix of pairwise distances estimated using the Maximum Composite Likelihood (MCL) approach, followed by the selection of the topology with the highest log likelihood value. The analysis involved 48 nucleotide sequences, with all positions containing gaps or missing data removed, resulting in a final data set of 1023 positions. The tree was oriented by using the 18S rRNA sequence of *Vairimorpha necatrix* Kramer, 1965 (Y00266) as the outgroup.

## 3. Results

### 3.1. Description of the Infection and Morphology of Microsporidia

The cysts detected in sardines exhibited morphological characteristics consistent with structures formed in fish parasitized by Microsporidia [37,38]. However, their morphology and presence in a new host (*Sardina pilchardus*) did not allow direct identification after literature and taxonomic review.

Each infected fish contained 2–10 cysts, ranging in size from 5 to 20 mm. These cysts were fragile, rupturing with minimal pressure or incision. Dissection under a stereoscopic microscope confirmed the absence of internal compartments and revealed that the cysts contained isolated microsporidian spores. The spores were spherical to ovoid (Figure 2A), likely varying in shape depending on their maturity stage. No extrusion of polar filaments was observed in vivo under light microscopy.

### 3.2. Xenoma

The morphology and location of the observed cysts, allow the differentiation between Xenoparasitic complexes and Xenoma. No variation in development was observed among the cysts analyzed using multiple microscopy techniques. The outer layer consisted of a fibroblasts envelope (connective tissue) approximately 40–50 μm thick, surrounding the spores.

TEM analysis of the cysts revealed both mature and immature spores, which were regularly shaped but not densely packed. These findings align with the definition of Xenoma proposed by Vávra and Larsson as “*a structure externally covered by a multilayered coat into which fibroblasts and epithelial fragments are incorporated*” [39].

### 3.3. Spores

**Figure 3:**
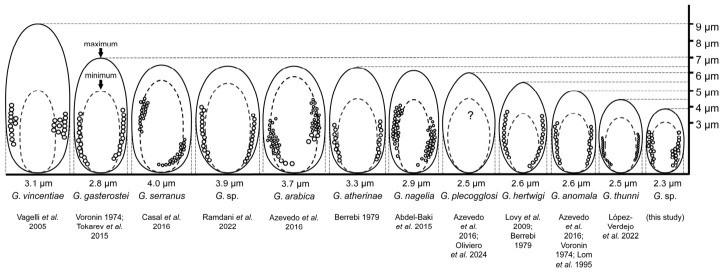
Schematic representation of spore size and morphology for *Glugea* species closely related to *Glugea* sp. obtained from sardines [11,16,37,41,42,43,44,45,46,47,48]. The question mark in the species *G. plecoglossi* indicates that no reliable information has been found regarding the number of coils or its size.

The spores were ovoid to pyriform (Figure 2A), similar to those of many other microsporidian species (Table 2, Figure 3), measuring 2.8–3.9 μm long and 1.8–2.3 μm wide. They possessed a dense, hard wall that was not penetrated effectively by osmium during TEM fixation, appearing as a white area surrounding the spores. Due to the hardness of the spores, some were displaced during cutting.

The polar tubes were isofilar type, with similar diameters, consisting of 6–7 wide coils and 10–15 coils arranged in 1–3 rows. The internal coil structure matched the description of Vávra and Larsson [39], exhibiting the six layered polar filament structure for both tube diameters (Figure 2B).

### 3.4. Molecular Characterization and Phylogeny

The alignment of the complete 18S rRNA (SSU) and partial 23S rRNA (LSU) coding regions for this new microsporidian species resulted in a consensus sequence of 1850 nucleotides and 50.9% GC content. This sequence was deposited in GenBank under accession number KY882286.

**Figure 4:**
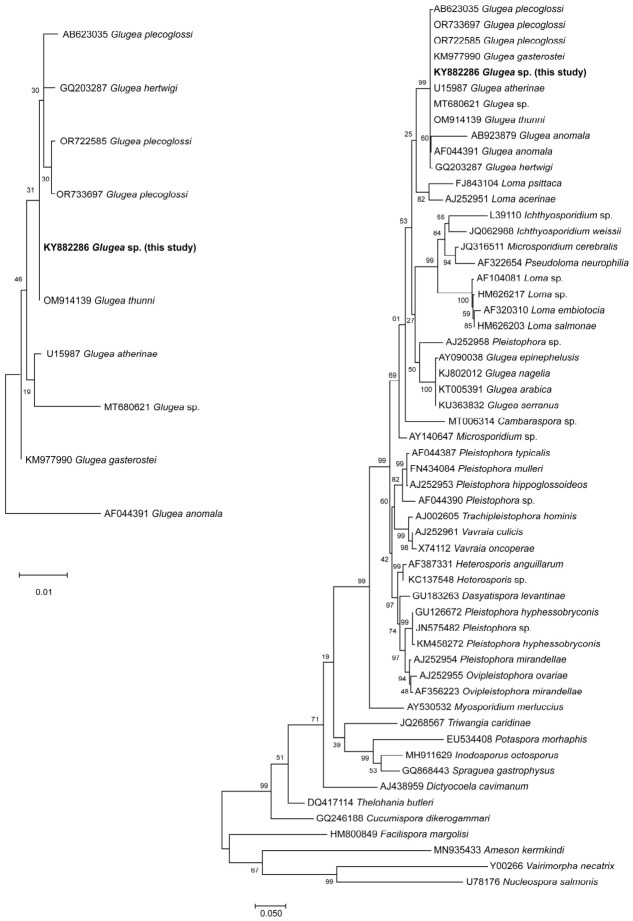
Maximum likelihood tree showing the relationship between *Glugea* sp. described in this study (KY882286) and other Microsporidia based on SSU RNA sequences. The bootstrap consensus tree inferred is taken to represent the evolutionary history of the taxa analyzed. Numbers on branches indicate bootstrap support values from 10,000 replications. Scale bar represents genetic distance. The tree was oriented by using the *Vairimorpha necatrix* (Y00266) as the outgroup. GenBank accession numbers are shown.

The phylogenetic analysis performed with SSU sequences placed this microsporidium within a clade with a bootstrap of 99% composed of *G. atherinae*, *G. thunni*, *G. plecoglossi*, *G. gasterostei*, *G. hertwigi* and *G. anomala* (Figure 4). Pairwise comparisons among these phylogenetically related organisms and other Microsporidia are presented in Table 3. These figures of genetic distance revealed a significant similarity of the isolated organism with *G. atherinae* (0.000), *G. thunni* (0.000), *G. plecoglossi* (0.001), *G. gasterostei* (0.001), *G. hertwigi* (0.002) and *G. anomala* (0.015) with other organisms showing a similarity lower than 0.915.

### 3.5. Taxonomic Summary

Phylum Microsporidia Balbiani 1882.

- Class Haplophasea Sprague, Becnel and Hazard 1992.
- Order Glugeida Issi, 1986.
- Family Glugeidae Thélohan, 1892.
- Genus *Glugea* Thélohan, 1891.
- Species *Glugea* sp.
- Host: European sardine, *Sardina pilchardus* Walbaum, 1792.

Description: Mature mononucleated spores measured 2.8–3.9 μm in length and 1.8–2.3 μm in width, while immature spores were spherical, ranging 1.8–2.2 μm in length. The isofilar polar tube measured 90–100 nm in width and contained 10–15 coils arranged in 1–3 rows.

The xenoma presents as a large, white, soft cyst surrounded by fibroblasts, located inside the visceral cavity, measuring 5–20 mm in length. The sardine specimens were part of a shipment in which a high prevalence of infection was observed, prompting further investigation to determine the identity of the microorganism.

Material studied: Histological sections (6 slides), a paraffin block, an Epon-embedded sample for TEM, and cysts preserved in formaldehyde have been deposited in the Museum of Zoology, University of Navarra, Spain, catalogue number MZ-20120425.

18S-23S rRNA sequence: GenBank accession number KY882286.

## 4. Discussion

A microsporidian species, *Glugea* sp., is herein described following its detection in the visceral cavity of commercial sardine (*Sardina pilchardus*). *Glugea* is the best-represented genus of Microsporidia infecting fish, occurring in both marine and freshwater species [49]. The morphological and ultrastructural features mainly used for the classification of Microsporidia include the number of nuclei (one or two), the number and size of spores, and the number of coils in the polar filament. The characteristics observed in the studied organism, especially in relation to the disposition of the spiral filament (row number), differ from those of other species in the genus, with the exception of *G. vincentiae* [11] (Table 2). Although *G. vincentiae* was reported in fishes from Port Phillip Bay, Southern Australia, its description lacked molecular data. A sample of stored *G. vincentiae* tissue was requested to perform molecular analysis, but no sequence could be obtained, likely due to inadequate preservation of the cysts.

The spores observed in sardines are morphologically similar to those of *G. vincentiae* in terms of number, arrangement, and type of polar tube coils. However, notable differences exist in spore dimensions: the present species exhibits spores measuring 2.8–3.9 μm in length × 1.8–2.3 μm in width, while *G. vincentiae* presented two types of spores, microspores with mean dimensions of 5.1 × 2.2 μm, and elongated oval macrospores (about 1% of spores), with mean dimensions of 8.9 × 3.1 μm. The xenoma found in sardine was located in the visceral cavity, in contrast to *G. vincentiae*, where the xenoma was found over the body surface, including the ventral region of the mandible, and the caudal and dorsal fins. The size of the cyst differs markedly: reaching up to 20 mm in the species found in sardines, compared to 1–2 mm in *G. vincentiae*. Regarding the host and geographic distribution of both *Glugea* species, there are differences as the apogonid (cardinal fish) *Vincentia conspersa* Klunzinger, 1872 infected by *G. vincentiae* is a marine species present in southern Australia (Melbourne coast), Tasmania and Flinders Island of northern Tasmania.

*Glugea atherinae*, *G. hertwigi*, *G. gasterostei, G. anomala* and *G. thunni* spores exhibit morphological similarities to the species identified in sardine, though they typically display a single row of polar tube coils and larger spore sizes: only *G. vincentiae* shares with the new species the arrangement of the filaments in more than one row. In the case of *G. gasterostei*, comparative studies revealed minimal differences from other species, leading some to consider it equivalent to *G. anomala* [1,9]. However, distinctions in xenoma formation–*G. anomala* producing xenomas beneath the skin versus *G. gasterostei* forming them in internal tissues (abdominal cavity according to the photograph of the report) [44]—have supported their acceptance as separate species.

Closely related species to the organism found in sardines in this study are *G. plecoglossi* and *G. thunni*, which show the highest similarity to this microsporidium based on molecular data (99.9–100%). Nevertheless, several morphological and ultrastructural differences existed according to the spore shape, size, number of rows and type of polar tube coils (Table 2). The size of the spores is a characteristic regularly used for the morphological differentiation of species. However, overlapping exists between spore dimensions (considering the minimum and maximum reported sizes by different authors) for the species under discussion (Figure 3). There is an active debate on whether all these organisms may belong to the same species, because of their morphological similarities and the small molecular distance among them, which does not completely clarify their identity [47].

The phylogenetic analysis placed *Glugea* sp. close to the clade composed of *G. hertwigi* (actually *Ichthyosporidium hertwigi*), *G. gasterostei*, *G. atherinae*, *G. plecoglossi* and *G. thunni*, with sequence differences ranging from 0.00% to 0.03% in the 18S rRNA. As discussed above, the morphology of *G. hertwigi* spores is different from the organism found in sardine [37].

Microsporidian taxonomy relies on an integrative framework combining morphological and genetic characteristics. Although molecular markers such as SSU rRNA are widely used, their high level of conservation can mask species-level differences. In fact, sequence similarities above 99% have been reported among taxa that differ in morphology, host range, and pathogenicity [17]. Moreover, consistent differences in spore morphology—particularly polar filament arrangement—together with ultrastructural traits and host specificity, have been shown to provide robust evidence for species delimitation even in the presence of minimal genetic divergence [50,51]. In this context, although the complete intracellular developmental cycle could not be characterized in the present study, the combination of morphological, ultrastructural, ecological, and molecular evidence supports that this *Glugea* sp. represents a distinct species. However, because its full developmental cycle could not be observed—and therefore could not be documented using TEM—no new species name is proposed.

It is important to highlight the relatively close genetic relationship with other Microsporidia known to cause human health problems such as *Pleistophora* and *Trachipleistophora* [52,53,54]. Moreover, the polar tube of *Glugea* has been found to be similar in composition and function to that of human pathogens belonging to the genus *Encephalitozoon* [55]. However, although no cases of human disease caused by *Glugea* have been reported, the precautionary principle warrants minimizing the exposure of immunocompromised individuals (e.g., AIDS, cancer, or transplant patients) to organisms such as microsporidia, particularly to *Glugea* species that are genetically close to those recognized as human pathogens [56]. This caution is further supported by ongoing discussion regarding host specificity, with some evidence suggesting that these microorganisms may be transmitted from invertebrates to mammals through adaptation to temperature changes [57].

*Glugea* species are often associated with crustacean hosts, many of which inhabit the pelagic zone. In this context, several authors have reported associations between Microsporidia and crustaceans of the genus *Gammarus* [58]. Given that sardines feed on cladocerans, diatoms, decapod larvae and copepods present in plankton [59], the microsporidian species described here may have the potential for a broad geographic distribution, considering the wide dispersal of its host and trophic interactions.

As with most *Glugea* species, the xenoma produced by the organism extracted from sardine is located in the visceral cavity, with only *G. vincentiae* causing small subcutaneous xenomas. The xenoma in sardine is large, contains many mature spores, and is encased in a very fragile layer, making it prone to rupture. Consequently, any manipulation of the visceral tissue can result in the widespread dispersal of spores, leading to the contamination of the animal’s body, as well as of hands, work surfaces, and instruments used during processing.

In relation to fish health, *Glugea* species have historically been associated with the collapse of commercial fisheries [60], like that caused by *G. hertwigi* in a fishery of rainbow smelt (*Osmerus mordax*) in New Hampshire [61]. Leading experts in the phylum *Microsporidia* have emphasized that the study of Microsporidia causing xenomas in fish is of particular interest, as many of these species are important agents of diseases in commercial fisheries [62]. A recent mass mortality event of round *Sardinella* has been reported, associated with a microsporidian infection caused by an organism closely related to *G. thunni*, but ultrastructurally distinct from *Glugea* sp. [63]. In addition, growing evidence shows that global warming is intensifying major fish diseases in aquaculture, making outbreaks more frequent and harder to manage [64].

The European Union regulates the acceptance of raw materials contaminated with parasites and classifies fishery products not suitable for human consumption as a “potential biological threat” [65,66]. In contrast, regulations in the United States do not consider any parasite in sardines as a threat [67]. Therefore, the situation described may represent a potential concern for both sardine populations and, under specific conditions, human health [68].

Eight genera of Microsporidia have been reported to infect humans: *Anncaliia*, *Encephalitozoon*, *Enterocytozoon*, *Microsporidium*, *Nosema*, *Pleistophora*, *Trachopleistophora* and *Vittaforma*. A wide variety of infections have been linked to Microsporidia, including myositis in skeletal muscle, chronic diarrhea, various systemic and respiratory diseases, and ocular infections [69,70,71,72,73,74]. Infections by Microsporidia have been increasingly diagnosed in immunosuppressed patients who undergo organ transplantation, and ocular infections have been reported in immunocompetent persons [75]. Their importance and frequency are increasing in recent years for two fundamental reasons: (1) increase in immunosuppressed patients, and (2) higher index of suspicion and better diagnostic tools. Therefore, incidence of Microsporidia infections could be greater than reported due to the existence of asymptomatic carriers among immunocompetent persons, the zoonotic nature of infections and the high presence in animals acting as reservoir [76].

## 5. Conclusions

This finding expands the known host range of *Glugea* and documents the emergence of a previously undescribed host–parasite association in a widely consumed marine species. The combined morphological, ultrastructural, and molecular evidence supports the consideration of *Glugea* sp. described in the present study as a species distinct from all previously described members of the genus. This parasite was found to infect the visceral cavity of the commercially important fish (*Sardina pilchardus*) from an Atlantic fishery. Given the economic and ecological relevance of sardines, the presence of xenoma-forming microsporidia warrants careful consideration, particularly in the context of fish health and food product quality. Moreover, although *Glugea* species have not been implicated in human disease, the recognized clinical importance of other microsporidia reinforces the need for vigilance, especially among immunocompromised populations. Overall, these results underscore the importance of integrative taxonomic approaches and support the relevance of a One Health perspective in addressing the potential implications of microsporidian infections at the interface of wildlife, food systems, and human health.

## Figures and Tables

**Table 1:** Amplification and sequencing primers for LSU and SSU in the *Glugea* samples studied.

Gene	Primer Name	Primer Sequence 5′–3′
LSU (23S rRNA)	NAP_99	AACAGRTCMGWKATGCCCT
	580R	GGTCCGTGTTTCAAGACGG
SSU (18S rRNA)	MICROF	CACCAGGTTGATTCTGCCTGA
	1492N_9	CTACAGCTACCTTGTTACGACTT
	MICRO_F426	CTGGTGCCAGCAYCCGCGG
	MICRO_R451	CATAGACACTCCAGGAGCTGG
	MICRO_F831	GACGGAAGAACACCACAAGGA
	MICRO_R875	GGTAAGCTGTCCCRCGTTGAG

**Table 2:** Morphological comparison of Microsporidia species related to *Glugea* sp. infecting sardine (this study) [11,15,16,37,40,41,42,43,44,45,46,47,48].

*Glugea* Species	Dif	Type	Rows	Number	Form	Long	Width	Host	Water	geo	loc	x-c	x-s	Ref	D
*G.* sp. (this study)	-	iso	3	10–15	o	2.8–3.9	1.8–2.3	*Sardina pilchardus* (Clupeidae)	S	AT	ac	w	10–20	-	-
*G.* sp.	0.000	iso	1	13–16	e, o	5.7–6.5	3.1–3.9	*Sardinella aurita* (Clupeidae)	S	AT	ac	w	6–15	[40]	5
*G. atherinae*	0.000	iso	1	10	s (1)	4.5–6.5	2.6–3.3	*Atherina boyeri* (Atherinidae)	S	FM	ac	w	1.5–13	[41]	6
*G. thunni*	0.000	iso	1	13–14	e, o	3.6–4.5	2.0–2.5	*Thunnus thynnus* (Scombridae)	S	SM	ac	w	0.2–7.5	[42]	6
*G. plecoglossi*	0.001	U	U	U	p	U	U	*Sardinella aurita* (Clupeidae)		IM	ac	w	1–5	[43]	4
*G. gasterostei*	0.001	iso	1	15–16	n, o	4.8–6.0	2.1–2.8	*Gasterosteus aculeatus* (Gasterosteidae)	S	FI	ac, c	y, w	5–6	[44,45]	7
*G. hertwigi* (2)	0.002	iso	1	12–13	p	3.5–5.5	1.5–2.6	*Osmerus eperlanus*, *O. mordax* (Osmeridae)	S	HO	ac	w	1–4	[37,41]	6
*G. plecoglossi*	0.003	U	U	U	e	5.1–6.2	2.0–2.5	*Plecoglossus altivelis* (Plecoglossidae); *Oncorhynchus mykiss* (Salmonidae)	S, F	JP	ac	w	2–3	[16]	5
*G. anomala*	0.015	iso	1	11–16	o	3.5–5.1	2.1–2.6	*Pungitius pungitius* (Gasterosteidae); *Nothobranchius eggersi*, *N. korthausae* (Cyprinodontidae), *Cynolebias nigripinnis* (Rivulidae), *Fundulopanchax filamentosus* (Nothobranchiidae)	S, F	AR	sc, ac	w	1–4	[16,44,46]	6
*G. vincentiae*	-	iso	1–3	12–14	o, n	5.1–8.9	2.2–3.1	*Vincentia conspersa* (Apogonidae)	S	US	sc	w	1–2	[11]	6
*G. arabica*	0.044	iso (3)	3–4	27–29	e, p	5.9–6.6	2.9–3.7	*Epinephelus polyphekadion* (Serranidae)	S	RS	iw	w, b	1	[16]	8
*G. serranus*	0.050	iso	2–3	18–19	e	6.5	3.5	*Serranus atricauda* (Serranidae)	S	MA	c, ac	w	10	[47]	5
*G. nagelia*	0.051	iso	3	26–29	o, p	4.3–6.0	1.8–2.9	*Cephalopholis hemistiktos* (Serrranidae)	S	RS	iw	w	2	[48]	6

(1) the size shows that is not “spheric”; (2) nowadays *Ichthyosporidium*; (3) the photograph in the description shows some differences between filaments (anisofilar); **dif**, (nucleotide pairwise distance); **type** (arrangement of filament of the spore): iso, isofilar; **rows**, number of filament rows into the spore; **number**, number of filament turns; **form** of spore: s, spheric; o, ovoid; e, ellipsoidal; p, pyriform; n, elongate. In our opinion, all spores are ovoid or ellipsoid, except for *G. plecoglossi* and *G. hertwigi*, which can be considered pear-shaped (Figure 3); **long** of spore: maximum length of the spore; **width** of spore, maximum width of the spore; **host**: species where has been found; **water**: S, sea water; F, freshwater; **geo** (geographical location of the reference): AT, Atlantic sea; JP, Japan; SM, Spain (Mediterranean); IM, Italy (Mediterranean); FM, France (Mediterranean); FI, Finland; HO, Holartic Region; AR, Arctic sea; US, USA; RS, Red sea; MA, Madeira (Portugal); **loc** (location of the infection): ac, abdominal cavity; c, connective; sc, subcutaneous connective; iw, intestine wall; **x-c** (xenoma colour): w, white-whitish; y, yellow; b, black; **x-s** (xenoma size): in mm; **ref**, reference; **D**, number of differences (not molecular) between *Glugea* sp. and the rest; U, unknown.

**Table 3:** Comparison of SSU rRNA sequences: percentage of identity (top diagonal) and pairwise distance (bottom diagonal) obtained by p-distance.

	1	2	3	4	5	6	7	8	9	10	11	12	13	14	15	16	17	18	19	20
1. KY882286 *Glugea* sp.		1.000	1.000	1.000	0.999	0.999	0.998	0.997	0.985	0.915	0.893	0.893	0.891	0.879	0.808	0.812	0.822	0.830	0.834	0.792
2. MT680621 *Glugea* sp.	0.000		1.000	1.000	0.998	0.999	0.997	0.996	0.984	0.914	0.893	0.893	0.891	0.878	0.808	0.811	0.822	0.829	0.834	0.793
3. U15987 *Glugea atherinae*	0.000	0.000		1.000	0.998	0.999	0.997	0.996	0.984	0.912	0.891	0.891	0.889	0.876	0.805	0.809	0.819	0.827	0.831	0.790
4. OM914139 *Glugea thunni*	0.000	0.000	0.000		0.998	0.999	0.997	0.996	0.984	0.914	0.893	0.893	0.891	0.878	0.807	0.811	0.821	0.829	0.834	0.792
5. OR722585 *Glugea plecoglossi*	0.001	0.002	0.002	0.002		0.997	0.997	0.996	0.985	0.913	0.884	0.884	0.882	0.868	0.821	0.823	0.808	0.817	0.821	0.781
6. KM977990 *Glugea gasterostei*	0.001	0.001	0.001	0.001	0.003		0.996	0.995	0.985	0.915	0.892	0.892	0.890	0.879	0.808	0.812	0.821	0.829	0.835	0.793
7. GQ203287 *Glugea hertwigi*	0.002	0.003	0.003	0.003	0.003	0.004		0.994	0.982	0.911	0.892	0.892	0.890	0.876	0.806	0.809	0.821	0.826	0.833	0.790
8. AB623035 *Glugea plecoglossi*	0.003	0.004	0.004	0.004	0.004	0.005	0.006		0.981	0.911	0.892	0.892	0.890	0.877	0.810	0.813	0.822	0.831	0.834	0.790
9. AF044391 *Glugea anomala*	0.015	0.016	0.016	0.016	0.015	0.015	0.018	0.019		0.935	0.885	0.885	0.883	0.868	0.800	0.804	0.815	0.822	0.830	0.792
10. AB923879 *Glugea anomala*	0.085	0.086	0.088	0.086	0.087	0.085	0.089	0.089	0.065		0.809	0.810	0.807	0.794	0.733	0.732	0.745	0.748	0.761	0.711
11. KT005391 *Glugea arabica*	0.107	0.107	0.109	0.107	0.116	0.108	0.108	0.108	0.115	0.191		0.998	0.999	0.862	0.826	0.834	0.828	0.832	0.831	0.794
12. KU363832 *Glugea serranus*	0.107	0.107	0.109	0.107	0.116	0.108	0.108	0.108	0.115	0.190	0.002		0.998	0.863	0.826	0.833	0.828	0.830	0.830	0.793
13. KJ802012 *Glugea nagelia*	0.109	0.109	0.111	0.109	0.118	0.110	0.110	0.110	0.117	0.193	0.001	0.002		0.862	0.825	0.832	0.827	0.830	0.829	0.792
14. AJ252951 *Loma acerinae*	0.121	0.122	0.124	0.122	0.132	0.121	0.124	0.123	0.132	0.206	0.138	0.137	0.138		0.811	0.823	0.839	0.826	0.843	0.758
15. JQ316511 *Microsporidium cerebralis*	0.192	0.192	0.195	0.193	0.179	0.192	0.194	0.190	0.200	0.267	0.174	0.174	0.175	0.189		0.899	0.770	0.767	0.782	0.730
16. JQ062988 *Ichthyosporidium weissii*	0.188	0.189	0.191	0.189	0.177	0.188	0.191	0.187	0.196	0.268	0.166	0.167	0.168	0.177	0.101		0.777	0.785	0.783	0.737
17. AJ252961 *Vavraia culicis*	0.178	0.178	0.181	0.179	0.192	0.179	0.179	0.178	0.185	0.255	0.172	0.172	0.173	0.161	0.230	0.223		0.878	0.953	0.820
18. GU126672 *Pleistophora hyphessobryconis*	0.170	0.171	0.173	0.171	0.183	0.171	0.174	0.169	0.178	0.252	0.168	0.170	0.170	0.174	0.233	0.215	0.122		0.875	0.817
19. AJ002605 *Trachipleistophora hominis*	0.166	0.166	0.169	0.166	0.179	0.165	0.167	0.166	0.170	0.239	0.169	0.170	0.171	0.157	0.218	0.217	0.047	0.125		0.824
20. AY530532 *Myosporidium merluccius*	0.208	0.207	0.210	0.208	0.219	0.207	0.210	0.210	0.208	0.289	0.206	0.207	0.208	0.242	0.270	0.263	0.180	0.183	0.176	

## Data Availability

The original contributions presented in this study are included in the article. 18S-23S rRNA sequence of the microorganism described has the GenBank-NCBI accession number KY882286.
